# *Salmonella* Phage X5 Alleviates *Salmonella pullorum*-Induced Growth Impairment in Chickens: Associations with Reduced Salmonella Burden, Modulated Intestinal Microbiome and Attenuated Inflammatory Responses

**DOI:** 10.3390/ani16203280

**Published:** 2026-10-17

**Authors:** Xinxin Jin, Qin Lu, Zhenggang Zhang, Ruiqin Liang, Tengfei Zhang, Qingping Luo

**Affiliations:** 1 Institute of Animal Sciences and Veterinary Medicine, Hubei Academy of Agricultural Sciences, Wuhan 430064, China; 2 College of Animal Science and Technology, Xinyang Agriculture and Forestry University, Xinyang 464000, China

**Keywords:** *Salmonella*, phage, chicken, replacing antibiotics, intestinal microbiota

## Abstract

*Salmonella* infection of chickens can cause persistent intestinal inflammation, intestinal microbiota disorder, and growth obstruction, which is an important bacterial disease causing significant economic losses in poultry farming. Phages, as an alternative to antibiotics as biological control agents, have advantages such as precise killing and no residue. This study aims to explore the effect and mechanism of the lytic *Salmonella* phage X5 in alleviating the growth impairment in chickens caused by *S. pullorum*. Oral administration of phages effectively prevents and controls *S. pullorum* infection in chickens. Phages can alleviate growth impairment caused by *Salmonella*, reduce *Salmonella* levels in the liver and ileum, lower serum concentrations of *IL-1β*, *IL-6*, and *TNF-α*, and decrease mRNA expression of inflammatory cytokines *IL-1β* and *IL-6* in the ileum. They also enhance mRNA expression of tight junction proteins *Claudin-3* and *ZO-1* in the ileal mucosa, helping maintain intestinal barrier integrity, and modulate the microbial community composition in the cecum. In the context of reduced and restricted antibiotic usage in livestock production, this study initially verified the protective effect of phage X5 against *Salmonella pullorum* infection in chickens, providing an experimental basis for the application of phages in the prevention and control of this bacterial disease, and offering new clues for analyzing the potential pathways through which bacteriophages confer host protective effects.

## Simple Summary

*Salmonella* is one of the most significant foodborne pathogenic bacteria worldwide. It is mainly transmitted through eggs, poultry meat, livestock meat, and dairy products, and is highly prone to causing collective food poisoning incidents due to cross-contamination during food processing. Phages, as a highly promising biological agent in the alternative control field, possess advantages such as strong host specificity, autonomous proliferation within the body, being unaffected by bacterial resistance phenotypes, short development cycle, low preparation cost, and ease of iterative optimization. This study evaluated the preventive and therapeutic efficacy of phage X5 on the infection of *Salmonella* in chickens in the evaluated system, providing theoretical support for the application of phages.

## 1. Introduction

*Salmonella* represents a critical foodborne pathogen for both humans and animals, capable of impairing host immunity and triggering intestinal as well as systemic disorders [1,2]. *Salmonella* can colonize the chicken gut and be transmitted to humans via contaminated eggs and poultry meat, thereby posing substantial threats to food safety and public health [3]. *Salmonella* in poultry is mainly transmitted vertically through the breeding eggs or horizontally through the digestive tract. *Salmonella* enterica serovar *Gallinarum* biovar *Pullorum* (*S*. *Pullorum*) causes pullorum disease, an infection largely adapted to poultry. Clinical manifestations of *S. pullorum* infection vary with chicken age. The main clinical manifestations of *S. pullorum* infection were head and neck retraction, fluffy feathers, decreased appetite, white feces, and anal paste in chicks within 2 weeks of age. In laying hens, this infection mainly damages the reproductive system, resulting in emaciation, reduced egg production and soft-shelled eggs. In addition, this pathogen can spread vertically through eggs and substantially reduce egg hatching rates [4,5].

Antibiotics are often used to control *Salmonellosis* in clinical practice, but the abuse of antibiotics easily leads to bacterial resistance and dysbiosis, aggravation of the difficulty in the prevention and control of bacterial diseases, and long development cycle and high cost of new antibiotic drugs [6,7]. Accordingly, novel antibiotic alternatives are urgently required to improve the prevention and therapy of bacterial diseases. Bacteriophages, viruses that selectively infect and lyse bacteria, represent one promising candidate. Compared with antibiotics, phages have the advantages of strong specificity, strong proliferation ability, no restriction of bacterial resistance, short development cycle, low cost, and easy renewal. Accordingly, bacteriophage-based strategies for preventing and treating bacterial infections have attracted substantial research attention [8,9,10]. *Salmonella* bacteriophage CKT1 effectively controls the vertical transmission of *S. pullorum* in young chickens [11].

To date, only a limited number of phages have been isolated and practically applied, and few studies have focused on phage-based strategies against *Salmonella pullorum* [12]. Therefore, this study employed a virulent *Salmonella pullorum* phage with a broad lytic spectrum that was screened from sewage samples collected from laying-hen farms; its biological characteristics have been reported in our previous study [13].

This study evaluated the preventive and therapeutic effects of phage X5 against *Salmonella pullorum* infection in chickens, aiming to explore a potential antibiotic-alternative candidate and provide experimental basis for further developing phage-based preventive strategies against *Salmonella pullorum* for poultry, and lay groundwork for future follow-up translational research.

## 2. Materials and Methods

### 2.1. Experimental Animal

SPF chicken embryos were purchased from Boehringer Ingelheim Viton Biotechnology Co., Ltd., Beijing, China. All hatched SPF chickens were mixed-sex from a single hatching batch. Chickens were housed in positive-pressure sterile isolator units. The rearing temperature was maintained at 34–35 °C from day 1 to day 3, then decreased by 1–2 °C every 48 h until reaching 26 °C, at which temperature it was held constant. Relative humidity was kept at 55–65%. Chickens were fed antibiotic-free, pathogen-free complete SPF chicken feed, with autoclaved water and feed provided ad libitum. The animal experimental protocol was approved by the Institutional Animal Care and Use Committee of Hubei Academy of Agricultural Sciences (approval number: HAAS-2023-0003). Ethics approval date: 9 March 2023.

### 2.2. Strains and Phage

*S. pullorum* CVCC519 was purchased from National Center for Veterinary Culture Collection (Beijing, China). For the CVCC519 strain, the minimum inhibitory concentration (MIC) of amoxicillin is 19.53 μg/mL, and the minimum bactericidal concentration (MBC) is 39.06 μg/mL. *S. pullorum* phage vB_SpuM_X5 (GenBank accession: PP502935) was deposited in our laboratory.

### 2.3. Design of Animal Experiments

In this experiment, 75 SPF chickens were randomly divided into 5 groups. Each group contained 3 independent isolator replicates, with 5 chickens per isolator (*n* = 15 chickens per group). Each isolator represents the primary experimental unit for the treatment factor; individual chickens within each isolator are sampling subunits.

**Figure 1:**
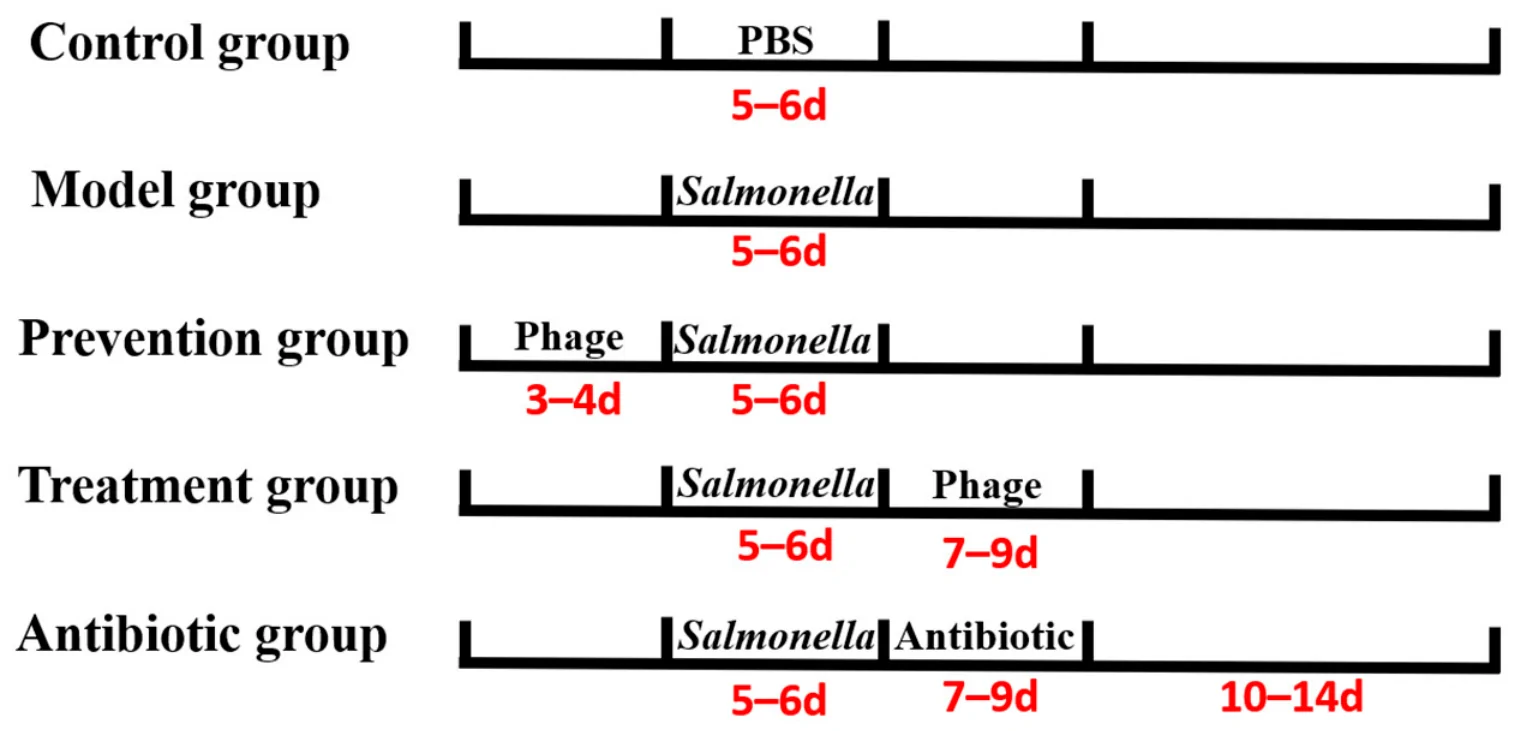
Grouping of animal experiments.

Group C (control group), four *Salmonella*-treated groups: group M (model group); group P (prevention group); group T (treatment group); group A (antibiotic group). *S. pullorum* challenge: *S. pullorum* CVCC519 was cultured in LB medium (37 °C, 180 rpm) to logarithmic-phase. Bacterial pellets were washed and resuspended in sterile PBS. Viable CFU counts of inocula were determined via plating on SS agar immediately prior to oral gavage. For all challenged groups (M, P, T, A), chickens received the challenge via oral gavage. Chickens in groups M, P, T and A were orally gavaged with 500 μL bacterial suspension containing 1 × 10^8^ CFU of *S. pullorum* on post-hatch day 5 and day 6 (two consecutive days). Group C received an equal volume of sterile PBS by gavage on day 5 and day 6. Group P: chickens received 500 μL phage suspension containing 10^9^ PFU phage on days 3 and 4 consecutively. Group T: 10^9^ PFU phage (500 μL) was administered continuously from day 7 to day 9. Group A: the same volume of 1 mg/mL Amoxicillin (500 μL) was continuously administered from day 7 to day 9 (Figure 1) [14]. Amoxicillin was delivered via oral gavage (same route as phage administration). The medication used during the amoxicillin group was commercial amoxicillin, with the veterinary drug code 120191199. Isolators housing different treatments were physically separated. The experimental animals had free access to feed and autoclaved drinking water. Each isolator served as the primary experimental unit for treatment allocation; individual chickens within one isolator were treated as sampling subunits. For statistical inference, isolator-level aggregated summaries were used where possible to avoid pseudoreplication arising from shared housing environment among co-housed chickens.

### 2.4. Detection Indices

#### 2.4.1. Survival and Final Body Weight

During the experiment, the health status of chickens was observed daily, clinical symptoms were recorded, and the number of deaths was recorded. All animal manipulations complied with institutional animal welfare guidelines. At the end of the experiment, the chicks were weighed after an overnight fasting. After confirming that the chicks were in a coma under general anesthesia, blood was collected and serum was separated. At the end of the experiment, blood was collected and serum was separated after confirming that the chicken was in a coma under general anesthesia.

#### 2.4.2. Immune Organ Weight and Organ Index

After slaughter, the spleen, thymus and bursa of Fabricius were weighed and the immune organ index was calculated. Organ index (%) = organ weight (g)/body weight (g) ×100.

#### 2.4.3. Bacterial Load in the Ileum and Liver

The liver and ileum of the chickens were placed in a centrifuge tube containing 1 mL pre-chilled PBS, and tissue weight was recorded. Tissues were homogenized, and homogenate fluids were serially ten-fold diluted. Then, 100 μL of each dilution gradient was spread onto SS solid medium and incubated at 37 °C overnight. Presumptive *S. pullorum* colonies were recognized based on typical morphological features (small, transparent colonies with black centers on SS agar). The analytical detection limit of this plating method was 100 CFU/g tissue. CFU per gram of tissue was calculated as: CFU/g = (countable colony number × dilution factor)/(tissue weight (g) × homogenate volume fraction) [15]. No phage-neutralization or inactivation steps were performed during sample processing; serial dilution was the sole method used to lower phage concentrations in plated aliquots.

#### 2.4.4. Determination of Serum Cytokines

At the end of the experiment, blood was collected and serum was separated after confirming that the chicken was in a coma under general anesthesia. Serum concentrations of tumor necrosis factor-α (*TNF-α*; BPE60016), interleukin-1β (*IL-1β*; BPE60017), and interleukin-6 (*IL-6*; BPE60049) from three samples per group were measured using an ELISA kit [16]. The kits were purchased from Shanghai Langton Biotechnology Co., Ltd., Shanghai, China and the operation was carried out in strict accordance with the ELISA kit operating instructions.

#### 2.4.5. Morphological Analysis of the Liver and Ileum

The fixed liver and ileum segments were dehydrated, embedded in paraffin, and sectioned for intestinal morphology and H&E stain [17]. Three chickens in each group were randomly selected, and a well-oriented tissue section was prepared for each chicken; For each chicken, ten intact, vertically oriented villus–crypt pairs per section were measured [18]. Morphometric measurements were completed by a researcher blinded to group allocation. For statistical analysis, the mean villus height and crypt depth for each individual chicken was calculated from all measured villi/crypts; these animal-level means served as statistical units rather than individual villus–crypt measurements. A Leica microscope (DM500, Leica, Wetzlar, Germany) was used to acquire images. CaseViewer 2.4 software was used to measure the villus height (Vh) and crypt depth (Cd) [19].

#### 2.4.6. 16S rRNA Gene Sequencing

The genomic DNA of microbial community was extracted from cecum content samples using intestinal microbial genomic DNA extraction kit (DP712-01, Tiangen, Beijing, China), following the manufacturer’s instructions. Primer 338F (5-ACTCCTACGGGAGGCAGCAG-3) and 806R (5-GGACTACHVGGGTWTCTAAT-3) were used to amplify the V3–V4 region of the bacterial 16S rDNA gene [20]. The purified PCR products were subjected to paired end sequencing (PE250) on the Illumina NovaSeq 6000 platform (Illumina Inc., San Diego, CA, USA). Firstly, Trimmomatic version 0.33 software was used to filter the sequenced Raw Reads. Then cutadapt 1.9.1 software was used to identify and remove primer sequences, and Clean Reads without primer sequences were obtained. The DADA2 method implemented in QIIME2 was used for denoising, paired-end read merging, and chimera removal to generate amplicon sequence variants (ASVs) [21,22,23]. Number of samples: report 6 sequencing samples per group. Alpha-diversity indexes including Chao1, Ace, Shannon and Simpson were calculated to reflect species richness and diversity. Gut–microbiota beta-diversity was analyzed using the Binary-Jaccard distance matrix, followed by principal coordinate analysis (PCoA) to visualize the microbial community structure across groups. Formal statistical testing of community composition differences among groups was performed by Permutational multivariate analysis of variance (PERMANOVA) (Binary-Jaccard distance). LEfSe was performed to identify differential abundant biomarkers. Briefly, the non-parametric factorial Kruskal–Wallis (KW) sum-rank test was used to detect features with significant differential abundance between classes of interest. Biological consistency was further validated by pairwise unpaired Wilcoxon rank-sum tests among subclasses. Finally, linear discriminant analysis (LDA) score threshold > 4.0 was applied to estimate the effect size of each differentially abundant feature.

#### 2.4.7. RNA Extraction from Ileum and Quantitative PCR

Total RNA was isolated from 0.1 g of ileal tissue using the TRIzol reagent (Takara Biotechnology Inc., Kusatsu, Shiga, Japan). Briefly, tissues were homogenized with autoclaved magnetic beads in a tissue homogenizer prior to RNA extraction. The microspectrophotometer NanoDrop One (Thermo Fisher Scientific, Madison, WI, USA) was used to assess RNA quality and concentration. A 260/280 absorbance ratio between 1.8 and 2.0 indicates acceptable RNA purity. One microgram of RNA was reverse-transcribed into cDNA using the cDNA Synthesis Kit (Takara Biotechnology). Quantitative real-time PCR for target genes and β-actin was performed using a Roche LightCycler 480 instrument (Roche Diagnostics, Basel, Switzerland). The primers of real-time PCR are presented in Table 1. Thermal cycling conditions were as follows: 95 °C for 30 s; 40 cycles of 95 °C for 5 s, 60 °C for 34 s; 95 °C for 15 s; 65 °C for 5 s; 95 °C for 5 s. The control gene was β-actin [24]. The mRNA expression levels of the inflammatory factors *IL-1β*, *IL-6*, and *IFN-γ* and tight junction proteins *Claudin-3*, *Occludin*, and *ZO-1* in the ileum were measured. Relative target-gene expression for each sample was calculated using the 2^−∆∆CT^ method [14].

### 2.5. Statistical Analysis

Data analyses were performed using GraphPad Prism 9.5 and R software (version 4.3.1). Considering our housing setup (3 independent isolators per treatment group with five chickens in each isolator), each isolator served as the primary statistical unit, whereas individual chickens within one isolator represented sampling subunits. To account for the nested experimental structure, two analytical strategies were applied: 1. For growth performance, organ indices, and tissue bacterial load endpoints: raw values from individual chickens within each isolator were averaged to generate one isolator-level summary value; these isolator-level means (*n* = 3 isolators per group) were then subjected to one-way ANOVA followed by post hoc multiple comparisons for treatment-level inference. For beta-diversity PERMANOVA (Binary-Jaccard distance, adonis2 from the R-package vegan), isolator was defined as a blocking variable. Permutations reshuffled complete sets of samples belonging to each isolator across treatment groups. This preserves the correlation between the two microbiome samples originating from the same isolator (sampling subunits), respects isolator as the primary experimental unit, and enables a valid statistical test for the treatment effect. The resulting PERMANOVA output was *R*^2^ = 0.38, *p* = 0.001. 2. For serum cytokine measurements, intestinal histomorphometry, qPCR gene expression, and 16S rRNA microbiota sequencing: a linear mixed-effects (nested) model was fitted, with treatment as the fixed factor and isolator nested within treatment as a random effect to account for non-independence of chickens housed in the same isolator. For endpoints with only one chicken sampled per isolator, the nested isolator random-effect term becomes non-identifiable and the model reduces statistically to one-way ANOVA; we kept the nested formula in code to explicitly reflect the hierarchical study design. For parametric analyses including one-way ANOVA and nested linear mixed-effects models, model residuals were diagnostically examined to verify key assumptions. Q-Q plots were used to evaluate residual normality, and residual-versus-fitted plots were inspected to assess homogeneity of variance. Tissue *Salmonella pullorum* CFU counts exhibited right-skewed distributions and were therefore log_10_-transformed prior to generating isolator-level summary values and statistical modeling. Levene’s test was additionally applied to confirm homoscedasticity. Where transformations could not satisfactorily stabilize variance, statistical outcomes were interpreted conservatively. As a permutation-based multivariate test, PERMANOVA does not require multivariate normality. Nevertheless, we ran the betadisper function to test for multivariate homogeneity of group dispersions. No significant difference in group dispersion was detected (*p* > 0.05), confirming that the observed PERMANOVA signal arose from true differences in microbial community composition rather than inter-group differences in within-group variability. All data are presented as mean ± SD. Significance thresholds were set as *p* < 0.05 (*), *p* < 0.01 (**), *p* < 0.001 (***), and *p* < 0.0001 (****).

## 3. Results

### 3.1. Effects of Phage on Survival Rate and Body Weight of Chickens Infected with Salmonella pullorum

**Figure 2:**
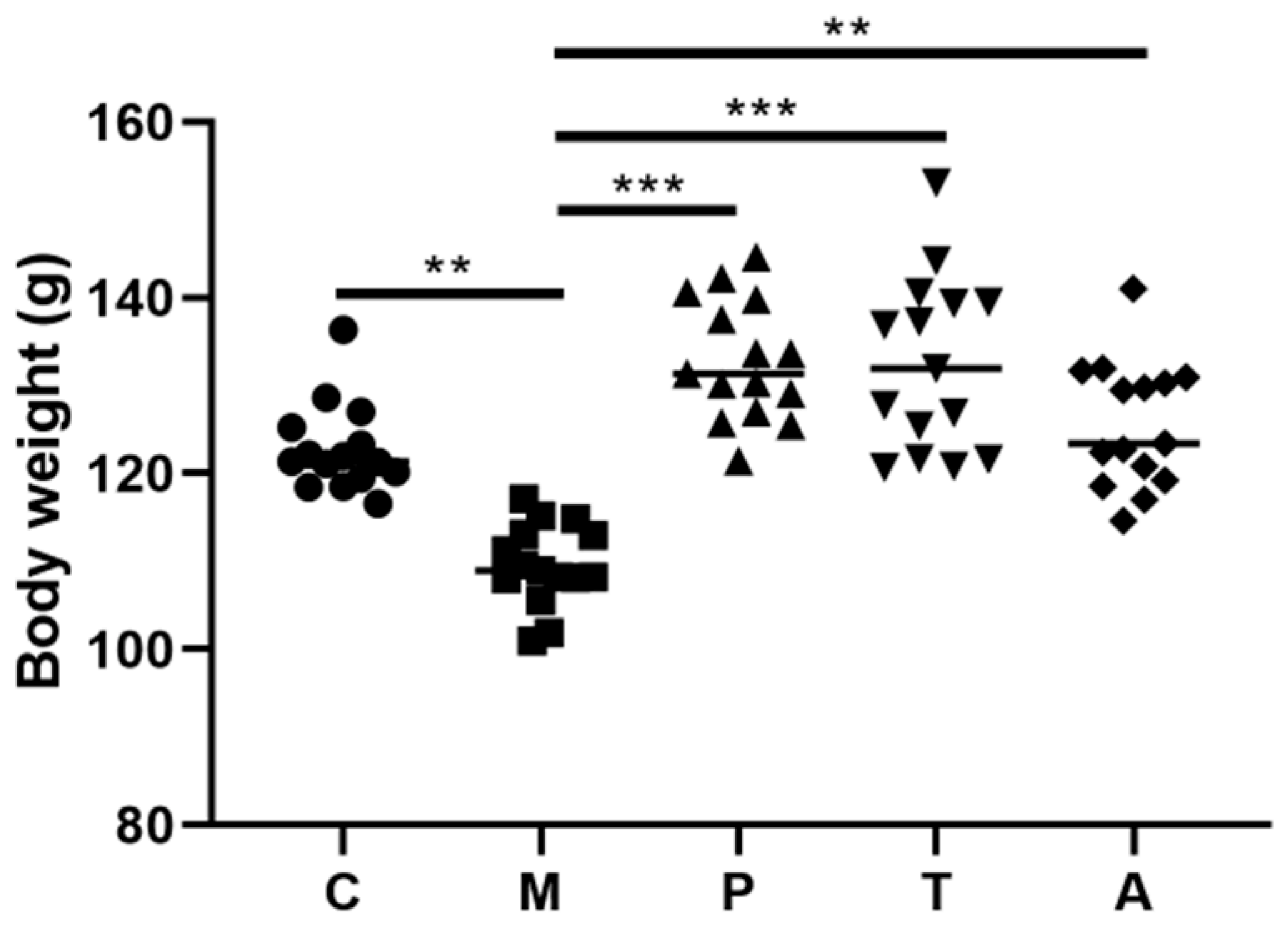
Effect of phage on body weight change in different groups of chickens. Each symbol shape represents an individual chicken sample, and different shapes denote different experimental groups. C, control group; M, model group; P, prevention group; T, treatment group; A, antibiotic group. Each symbol represents body weight from an individual chicken (sampling subunit). Error bars denote mean ± SD. Statistical inference was performed using one-way ANOVA on isolator-averaged body-weight values (*n* = 3 isolators per group, primary experimental unit), followed by Tukey’s HSD post hoc test. Significance thresholds were set as *p* < 0.01 (**), *p* < 0.001 (***).

All chickens survived throughout the *Salmonella pullorum* challenge experiment. In Figure 2, the infection of *S. pullorum* had a significant effect on body weight, and the body weight of group M was significantly lower than that of group C (*p* < 0.01). Body weights of groups P, T, and A differed significantly from those of group M (*p* < 0.01). The decreased final body weight observed in group M reflected growth retardation and compromised weight gain.

### 3.2. Effect of Phage on Immune Organs of Chickens Infected with Salmonella pullorum

**Figure 3:**
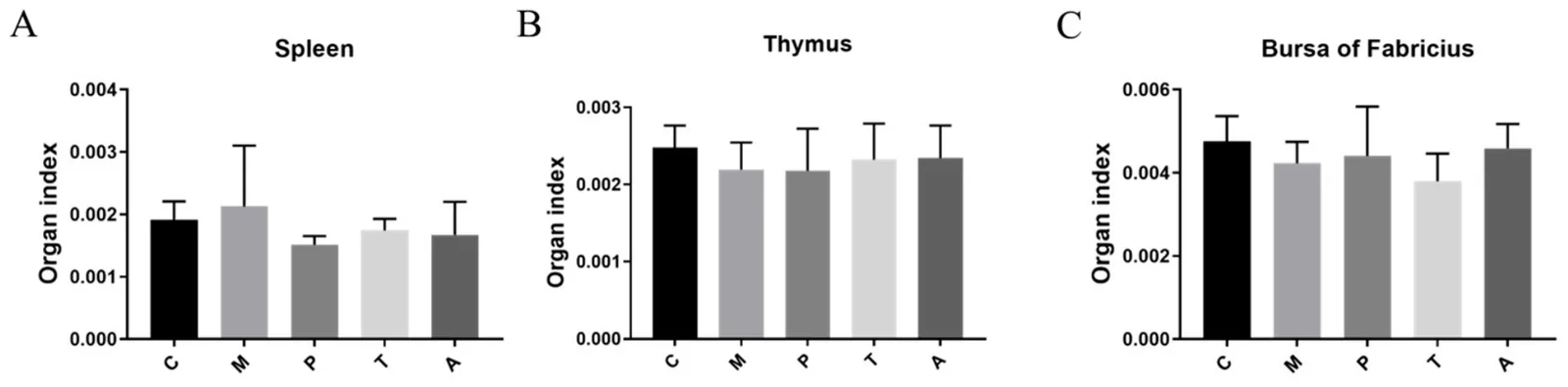
Effect of phage on immune organ index of different groups of chickens (**A**) Spleen index; (**B**) Thymus index; (**C**) Bursa of Fabricius index. C, control group; M, model group; P, prevention group; T, treatment group; A, antibiotic group. Each symbol represents data from an individual chicken (sampling subunit). Error bars denote mean ± SD. Statistical inference was performed using one-way ANOVA on isolator-averaged organ-index values (3 isolators per group, primary experimental unit), followed by Tukey’s HSD post hoc test.

No significant inter-group differences were observed for the organ indices of the spleen, thymus and bursa of Fabricius (Figure 3).

### 3.3. Effect of Phage on Tissue Load of Chickens Infected with Salmonella pullorum

**Figure 4:**
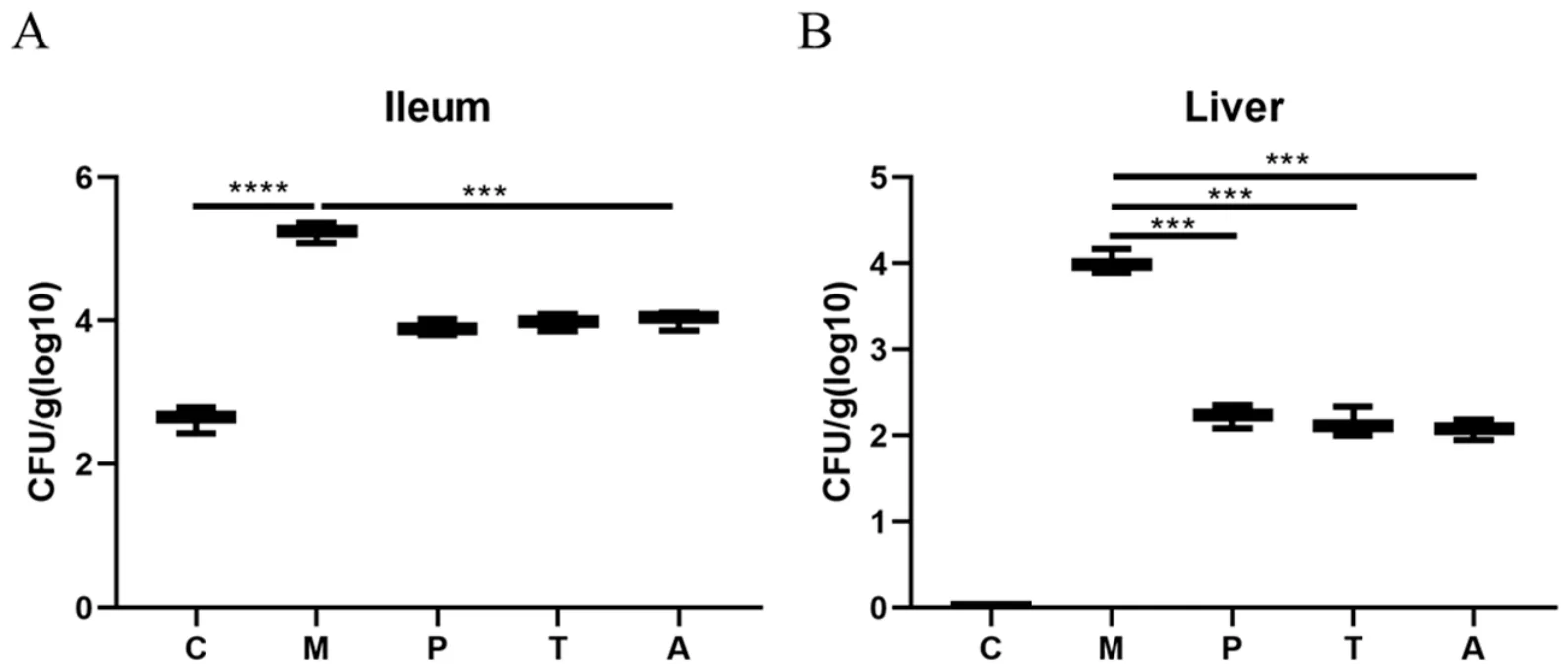
Effect of phage on tissue load of chickens infected with *Salmonella pullorum* (**A**) Ileal bacterial burden; (**B**) Hepatic bacterial burden. C, control group; M, model group; P, prevention group; T, treatment group; A, antibiotic group. Bacterial counts were log_10_-transformed prior to analysis. Error bars denote mean ± SD. One-way ANOVA was performed on isolator-averaged bacterial-load values (*n* = 3 isolators per group, primary experimental unit), followed by Tukey’s HSD post hoc test. Significance thresholds were set as *p* < 0.001 (***), and *p* < 0.0001 (****).

Compared with group M, the bacterial load of *Salmonella pullorum* in liver and ileum of chickens treated with phage X5 was significantly reduced (*p* < 0.05) (Figure 4). The body weight of the chickens treated with phage was significantly different from that of the infected chickens, and the phage treatment could reduce the *Salmonella pullorum* load in the ileum and liver.

### 3.4. The Effect of Phages on Serum Cytokines in Chickens Infected with Salmonella pullorum

**Figure 5:**
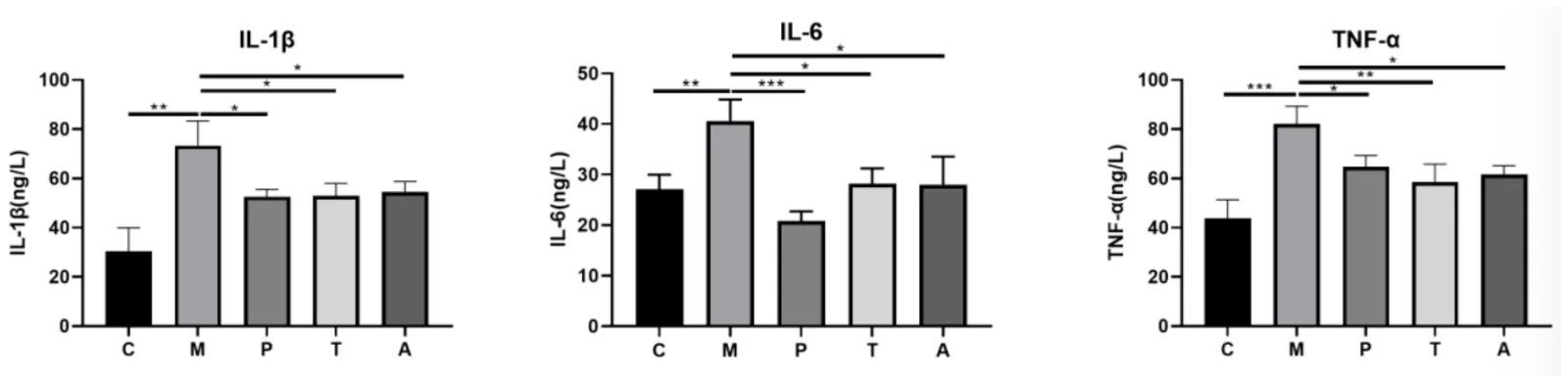
Effect of phage on inflammatory cytokines in serum of chickens C, control group; M, model group; P, prevention group; T, treatment group; A, antibiotic group. *n* = 3 chickens per group (one chicken sampled from each of the three isolators per group; sampling subunits). Error bars denote mean ± SD. A nested linear mixed-effects model was applied for statistical testing. Note that the nested random-effect term for isolators became non-identifiable when only one chicken was sampled per isolator; consequently, between-group comparisons reduced statistically to a one-way ANOVA. Significance thresholds were set as *p* < 0.05 (*), *p* < 0.01 (**), *p* < 0.001 (***).

Infection of chickens with *S. pullorum* in the intestinal tract can cause intestinal damage and may lead to changes in cytokines in the serum. Therefore, the contents of cytokines *IL-1β*, *IL-6* and *TNF-α* in the serum were detected. As shown in Figure 5, the contents of *IL-1β*, *IL-6* and *TNF-α* in group M infected with chicken white dysentery *Salmonella pullorum* were significantly higher than those in group C (*p* < 0.01). The *IL-6* contents in the phage prevention group P, phage treatment group T, and antibiotic group A did not show significant differences compared to group C, but there were significant differences compared to group M (*p* < 0.05). The contents of *IL-1β* and *TNF-α* in groups P, T and A were slightly higher than those in group C, and significantly lower than those in group M (*p* < 0.05). The infection of chickens with *Salmonella pullorum* has a significant impact on the cytokines in the serum. Both phages and antibiotics can reduce the contents of *IL-1β*, *IL-6* and *TNF-α*.

### 3.5. Effect of Phage on Intestinal Morphology of Chickens

**Figure 6:**
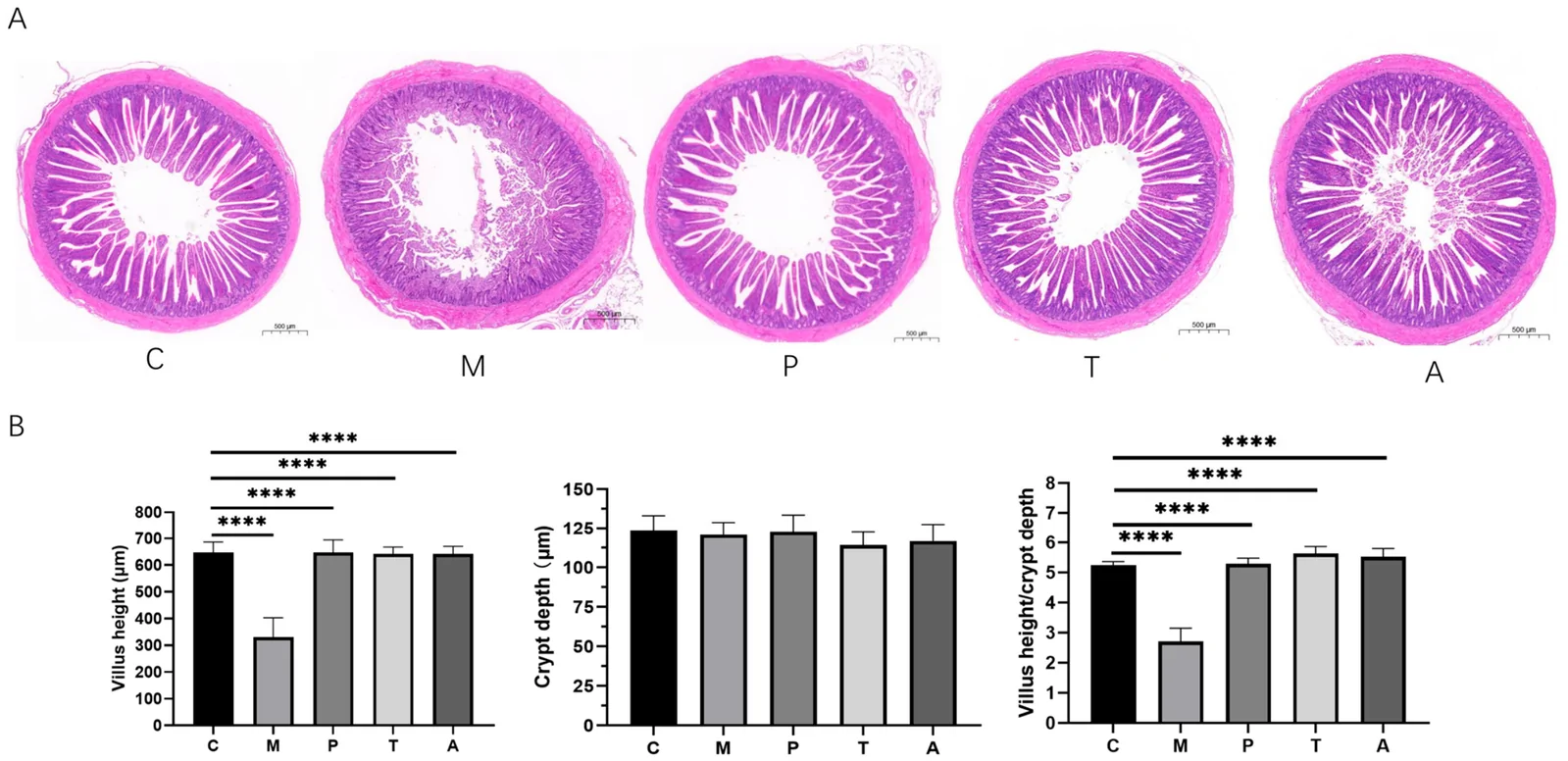
Effect of phage on the morphology of ileum of chickens (500 μm). (**A**) Representative H&E-stained ileum histology sections; (**B**) morphometric indices: villus height, crypt depth, villus-height-to-crypt-depth ratio. C, control group; M, model group; P, prevention group; T, treatment group; A, antibiotic group. *n* = 3 chickens per group (one chicken sampled from each of the three isolators per group; sampling subunits). Ten villus–crypt pairs were measured per chicken; chicken-level averaged morphometric values were used for analysis. Error bars denote mean ± SD. A nested linear mixed-effects model was applied for statistical testing. Significance thresholds were set as *p* < 0.0001 (****).

After HE staining, the height of the villi and the depth of the crypts in the ileum were measured to evaluate the histological morphology of the ileum (Figure 6). Ileal villi from the group C exhibited regular organization and intact morphology. In group M, ileal villi displayed disorganized, loosely arranged architecture with blurred contours. Mucosal crypt structures were abnormal, accompanied by irregular tissue accumulation, indicating severe intestinal injury. Compared with group M, the arrangement of intestinal villi in the phage-treated group was more regular, the outline was more complete, and the damage was less severe (*p* < 0.01). The differences in ileal villus morphology between the phage treatment group T and the antibiotic treatment group A were not significant. Both phage X5 and amoxicillin mitigated the reduction in villus height and villus-to-crypt (V/C) ratio induced by *Salmonella pullorum* infection (*p* < 0.01). These findings suggest that phage X5 alleviates ileal-villus injury triggered by *Salmonella pullorum* and preserves normal ileal tissue morphology.

### 3.6. Effect of Phage on Liver of Chickens

**Figure 7:**
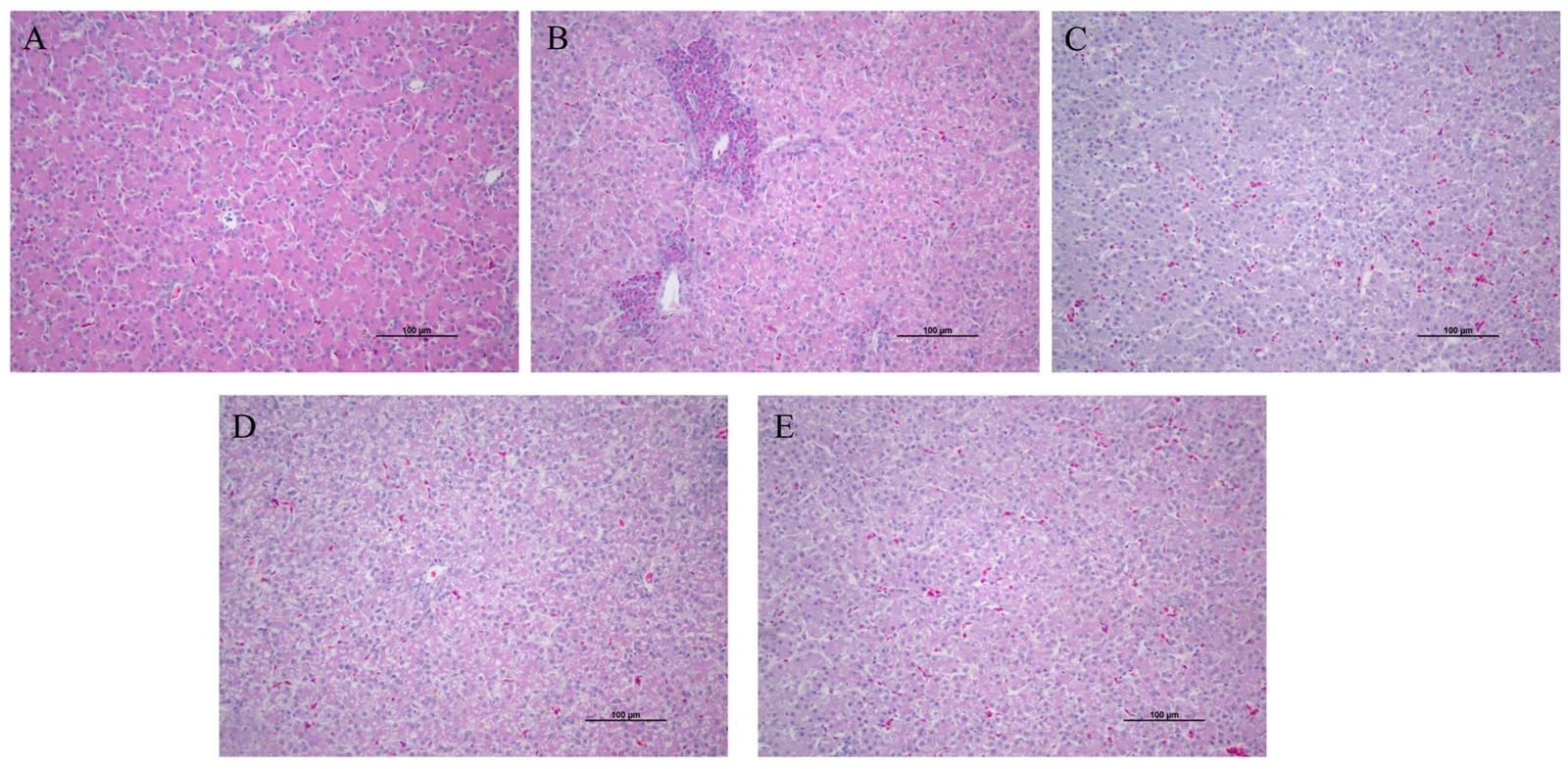
Effect of phage on the morphology of liver of chickens (500 μm). (**A**) Group C; (**B**) Group M; (**C**) Group P; (**D**) Group T; (**E**) Group A. C, control group; M, model group; P, prevention group; T, treatment group; A, antibiotic group. Representative histological images from *n* = 3 chickens per group (one chicken per isolator). Qualitative histopathological evaluation was performed; no quantitative statistical comparison was conducted for liver histology images.

The liver tissue of group C was uniformly stained, liver cell cytoplasm was abundant, morphological structure was normal, and no significant expansion of the liver sinuses was seen, nor was there any significant inflammation. Group M livers exhibited widespread sinusoidal dilation, extensive infiltration by lymphocytes and heterophils, as well as hepatocyte necrosis, nuclear fragmentation and karyolysis. The liver pathological damage of chickens in group P, group T and group A was relatively mild, with no obvious lesions observed (Figure 7).

### 3.7. Effect of Phage on the mRNA Levels of Cytokines and Tight Junction Proteins in the Ileum of Chickens

**Figure 8:**
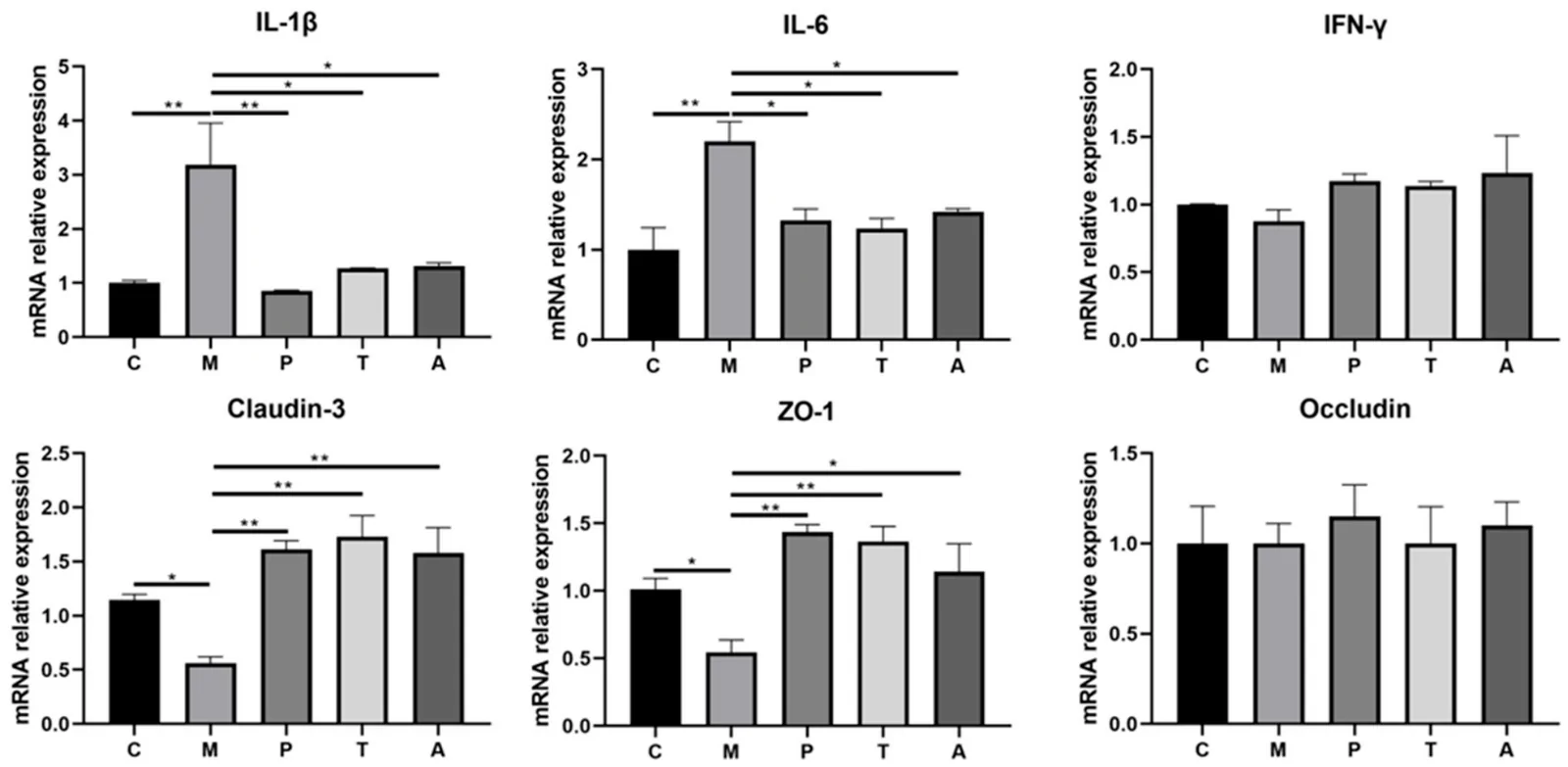
Effect of phage on mRNA expression levels of ileal cytokines and tight junction protein in chickens C, control group; M, model group; P, prevention group; T, treatment group; A, antibiotic group. *n* = 3 isolators per group. Error bars denote mean ± SD. Significance thresholds were set as *p* < 0.05 (*), *p* < 0.01 (**).

Phage treatment down-regulated the ileal mRNA expression of pro-inflammatory IL-1β and IL-6 induced by *Salmonella pullorum* infection (*p* < 0.05) (Figure 8). Compared with the model group, the phage treatment group T was able to increase the expression levels of the intestinal tight junction proteins Claudin-3 and ZO-1 (*p* < 0.05).

### 3.8. Effect of Phage on Cecum Microbiota of Chickens Infected with Salmonella pullorum

**Figure 9:**
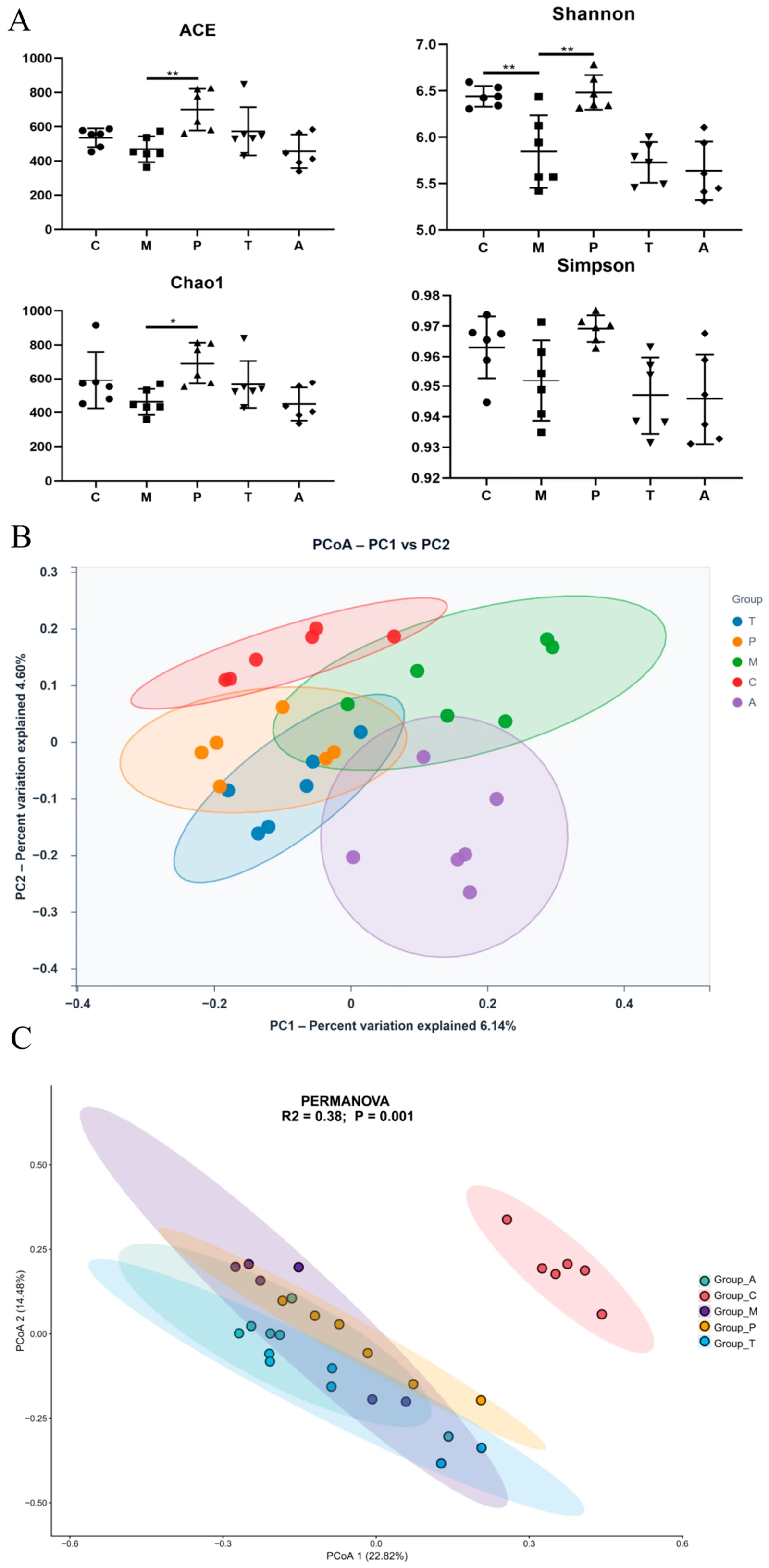
Analysis of Alpha and Beta diversity of the cecal microbiota in chickens. (**A**) Alpha diversity analysis of cecal microbiota in chickens. Each symbol shape represents an individual chicken sample, and different shapes denote different experimental groups. The values are means ± SD. Significance thresholds were set as *p* < 0.05 (*), *p* < 0.01 (**). (**B**) Beta-diversity PCoA ordination based on the Binary-Jaccard distance metric. PCoA provides descriptive visualization only. (**C**) Formal group-composition comparisons were performed using PERMANOVA (Binary-Jaccard distance), with permutations of complete isolator-level sample sets across treatments to respect isolator as the primary experimental unit. C, control group; M, model group; P, prevention group; T, treatment group; A, antibiotic group. *n* = 6 sequencing samples per group (2 chickens randomly sampled per isolator, 3 isolators per group).

The results of Alpha diversity analysis are shown in Figure 9A: The Ace index values differed significantly between group M and group P (*p* < 0.01); The Shannon index differed significantly between group C and group M (*p* < 0.01), as well as between group M and group P (*p* < 0.01); The Chao1 index differed significantly between group M and group P (*p* < 0.05); Simpson: there was no significant difference between groups.

The results of PCoA ordination (Binary-Jaccard distance) showed that cecal bacterial community points of group M appeared visually scattered, while samples from groups C, P, T, and A formed more-compact clusters (Figure 9B). PCoA based on Binary-Jaccard distance revealed obvious separation of microbial communities in the control group from other experimental groups (Figure 9C). The first and second principal coordinates explained 22.82% and 14.48% of the total community variance, respectively. PERMANOVA, implemented with permutation of full isolator-level sample sets across treatments, demonstrated that treatment significantly shaped gut microbiota composition ((*R*^2^ = 0.38), *p* = 0.001). Group C clearly separated from the other four groups, samples from M, P, T and A groups overlapped in the ordination space.

**Figure 10:**
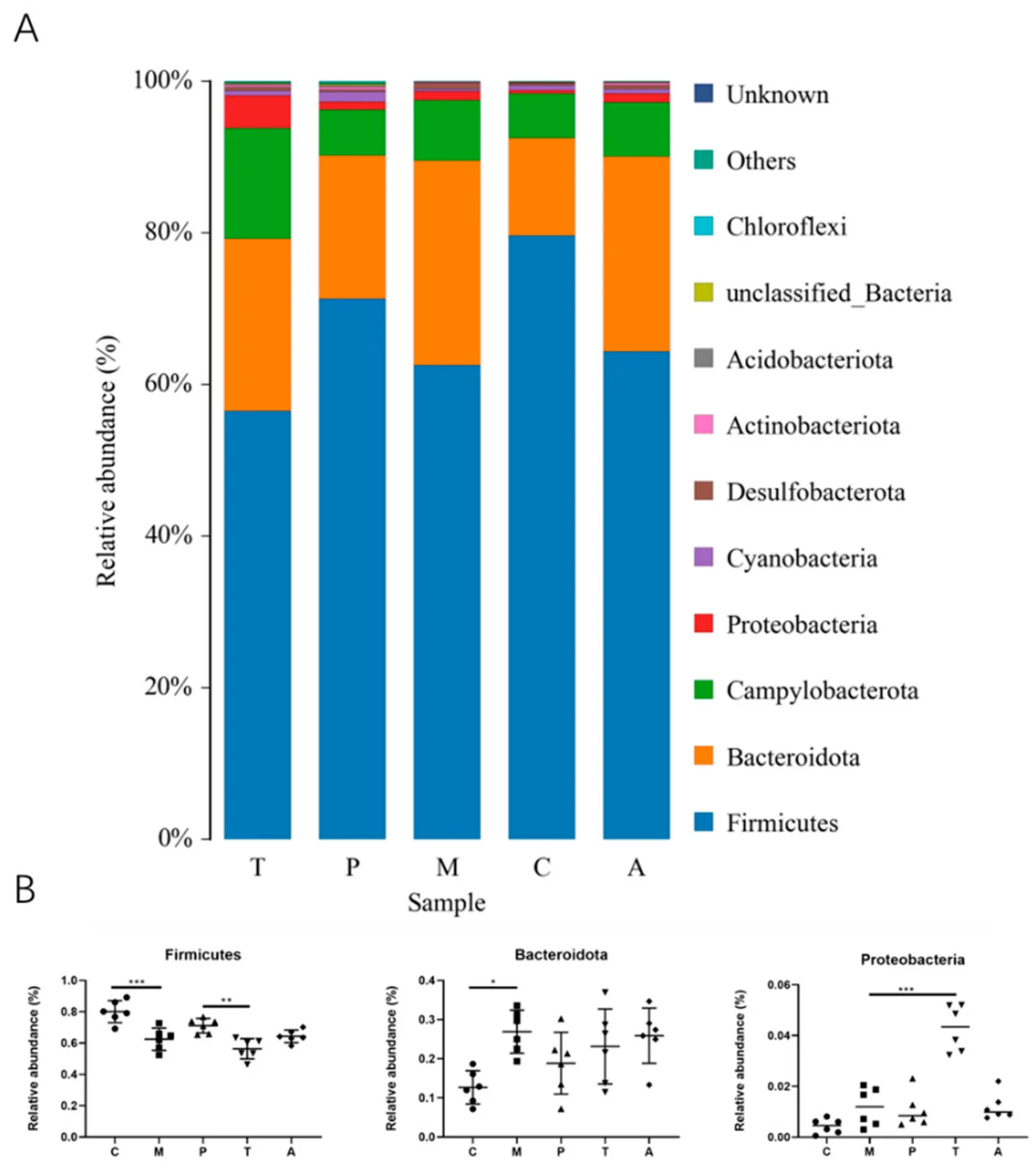
Analysis of species composition and diversity at the phylum level of the cecal microbiota. (**A**) Species composition at the phylum level of the cecal microbiota. (**B**) Analysis of the differences at the phylum level of the cecal microbiota. C, control group; M, model group; P, prevention group; T, treatment group; A, antibiotic group. *n* = 6 sequencing samples per group (2 chickens per isolator, 3 isolators per group). Error bars denote mean ± SD. Pairwise taxon-level comparisons were corrected for multiple testing using the Benjamini–Hochberg FDR procedure. Significance thresholds were set as *p* < 0.05 (*), *p* < 0.01 (**), *p* < 0.001 (***).

Phylum-level profiling of cecal microbiota revealed that Firmicutes constituted the predominant bacterial phylum, while Bacteroidetes phylum was the second dominant phylum (Figure 10). The average relative abundances of the Firmicutes phylum in groups C, M, P, T, and A were: 80.10%, 62.50%, 71.11%, 56.41%, and 64.39% respectively; the average relative abundances of the Bacteroidetes phylum were: 12.70%, 26.92%, 18.87%, 23.14%, and 25.90% respectively; and the average relative abundances of the Proteobacteria phylum were: 0.43%, 1.19%, 1.06%, 4.28%, and 1.20% respectively. The relative abundance of phylum *Firmicutes* was significantly higher in group M than in group T (*p* < 0.001); it was also significantly higher in group P relative to group T (*p* < 0.01); the abundance of the Bacteroidetes phylum in group M was significantly higher than that in group C (*p* < 0.05); the relative abundance of the Proteobacteria phylum in group M was significantly lower than that in group T (*p* < 0.001).

**Figure 11:**
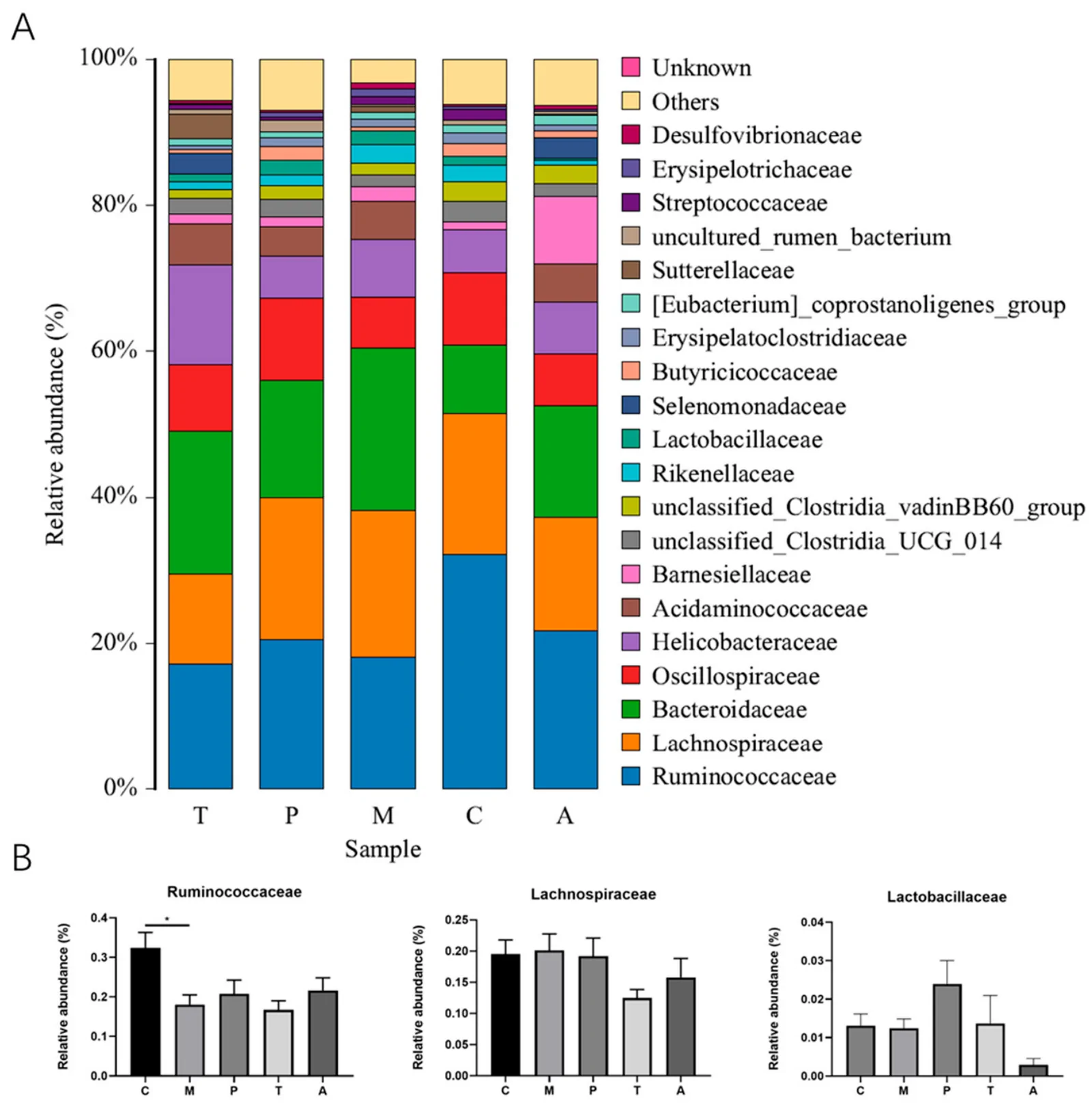
Analysis of species composition and diversity at the family level of the cecal microbiota. (**A**) Species composition at the family level of the cecal microbiota. (**B**) Analysis of the differences at the family level of the cecal microbiota, C, control group; M, model group; P, prevention group; T, treatment group; A, antibiotic group. *n* = 6 sequencing samples per group (2 chickens per isolator, 3 isolators per group). Error bars denote mean ± SD. Pairwise taxon-level comparisons were corrected for multiple testing using the Benjamini–Hochberg FDR procedure. Significance thresholds were set as *p* < 0.05 (*).

At the family level, the cecal microbiota analysis revealed that *Ruminococcaceae* was the dominant family, followed by *Lachnospiraceae* as the second most abundant family. The average relative abundances of the *Ruminococcaceae* family in groups C, M, P, T, and A were as follows: 32.40%, 18.03%, 20.80%, 16.71%, and 21.60% respectively; the average relative abundances of the *Lachnospiraceae* family were 19.54%, 20.11%, 19.19%, 12.50%, and 15.78% respectively; the average relative abundances of the *Lactobacillaceae* family were 1.21%, 1.87%, 2.05%, 1.16%, and 0.25% respectively (Figure 11A). The average relative abundances of the *Ruminococcaceae* family and the *Lactobacillaceae* family in group P were 20.80% and 2.05% respectively, which were higher than those in group M (18.03% and 1.87% respectively). At the genus level (Figure 11B), the relative abundance of the *Ruminococcaceae* family in group C was significantly higher than that in group M (*p* < 0.05). The relative abundances of the *Ruminococcaceae* and *Lactobacillaceae* families in group P were not significantly different from those in group M, but showed an increasing trend, which might be related to the differences among the samples; the relative abundance of the *Mucisphaerae* family in group M was higher than that in group T, but there was no significant difference, and there was also no significant difference compared with group P (Figure 11B).

**Figure 12:**
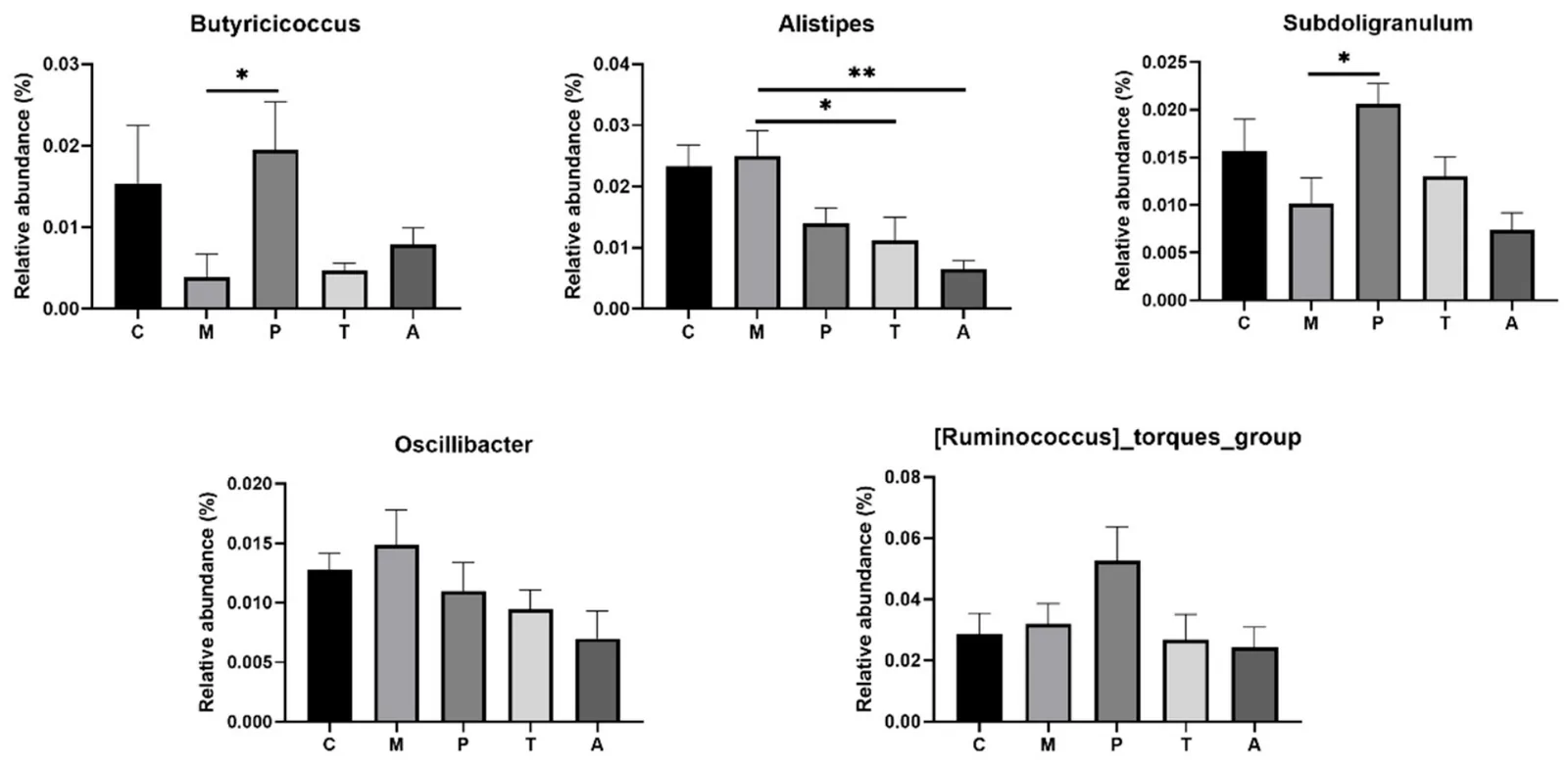
Analysis of species diversity at the genus level of the cecal microbiota. C, control group; M, model group; P, prevention group; T, treatment group; A, antibiotic group. *n* = 6 sequencing samples per group (2 chickens per isolator, 3 isolators per group). Error bars denote mean ± SD. Pairwise taxon-level comparisons were corrected for multiple testing using the Benjamini–Hochberg FDR procedure. Significance thresholds were set as *p* < 0.05 (*), *p* < 0.01 (**).

The genus-level results of the cecal microbiota showed that the relative abundance of the *Butyricicoccus* genus in group P was significantly higher than that in group M (*p* < 0.05); the relative abundance of the *Alistipes* genus in group T and group A was significantly lower than that in group M (*p* < 0.05); the relative abundance of the *Subdoligranulum* genus in group P was significantly higher than that in group M (*p* < 0.05); the relative abundance of the *Oscillibacter* genus in group P, group T, and group A showed a trend of decrease compared to group M, but no significant difference was observed; the relative abundance of the *Ruminococcus_torques*_group genus in group P showed a trend of increase compared to group M, but no significant difference was observed (Figure 12).

**Figure 13:**
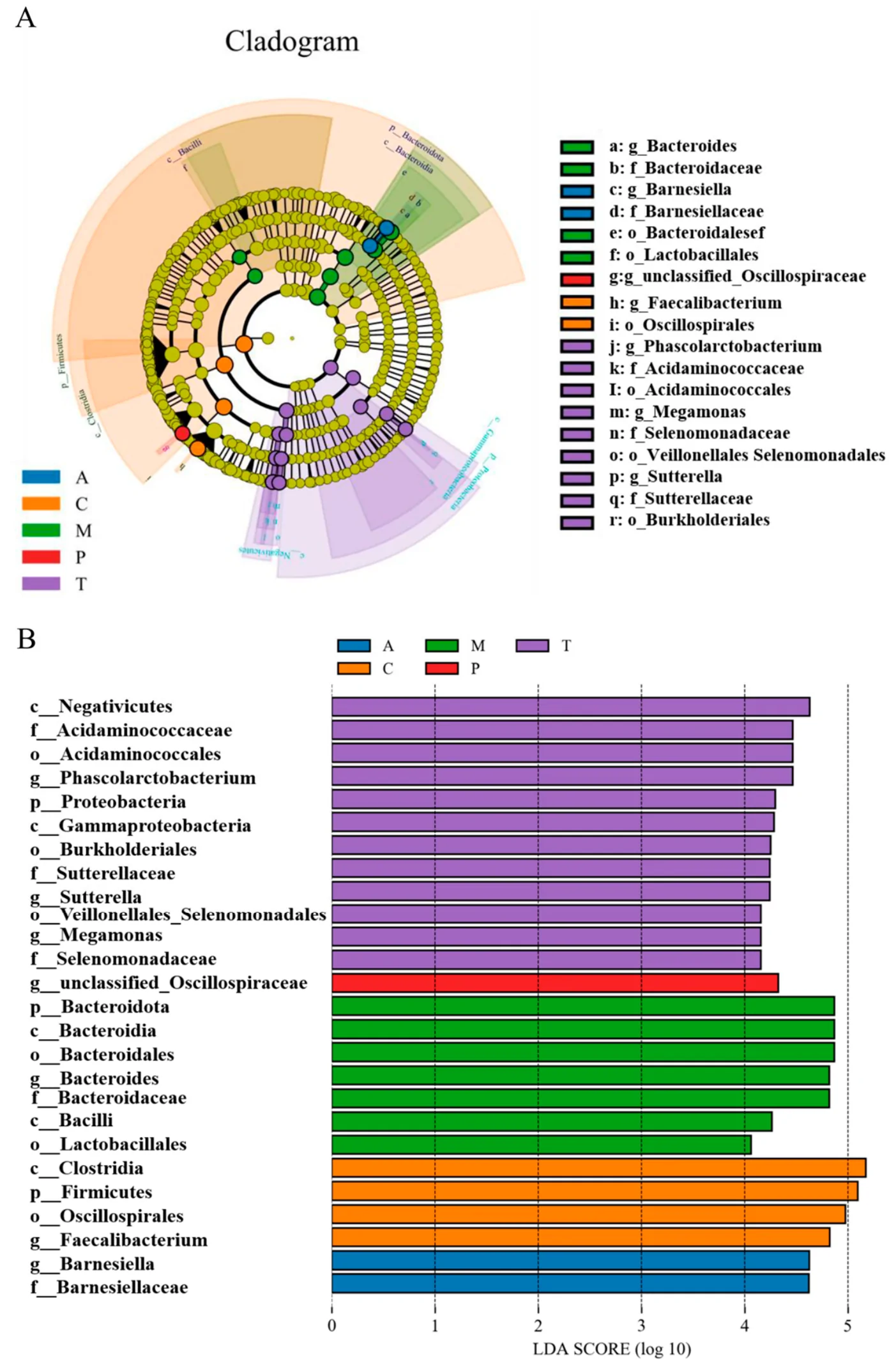
LEfSe analysis of cecal microbiota. (**A**) Cladogram; (**B**) LDA scores. Different-colored regions represent different constituents. Circles indicate phylogenetic level from phylum to genus. Taxa with LDA score > 4.0 are shown; multi-class comparison mode was set to one-against-all. LEfSe implements internal feature-level multiple-testing correction. The length of the histogram represents the LDA score. C, control group; M, model group; P, prevention group; T, treatment group; A, antibiotic group. *n* = 6 sequencing samples per group (2 chickens per isolator, 3 isolators per group).

LEfSe analysis was performed to identify cecal microbiota taxa that differed significantly among group C, group M, group P, group T and group A. The cladogram displayed the phylogenetic distribution of the differential taxa from phylum to genus level, in which colored nodes and outer-ring sectors denoted taxa significantly enriched in each group (Figure 13A). The effect size of each biomarker was quantified by the LDA score (log10) with a threshold of >4 (Figure 13B). In group C, the Clostridia within Firmicutes represented the strongest biomarker. In group M, taxa belonging to *Bacteroidota*, including *Bacteroidia*, *Bacteroidales*, *Bacteroidaceae* and *Bacteroides*, were markedly enriched, indicating characteristic gut dysbiosis following model establishment. In group A, *Barnesiella* and its family *Barnesiellaceae* were the signature taxa. Only one differential taxon (unclassified *Oscillospiraceae*) was identified in group P, indicating that prophylactic phage intervention caused minimal perturbation to gut microbiota. Group T showed the largest number of differential biomarkers, which belonged mainly to *Negativicutes* and *Proteobacteria*, including *Phascolarctobacterium*, *Megamonas*, *Sutterella* and *Burkholderiales*.

## 4. Discussion

Many countries have banned the use of antibiotics in animal feed as growth promoters, leading to a renewed focus on phage therapy [25]. Our previous characterization of phage X5 demonstrated its strong lytic activity, short latent period, as well as favorable pH tolerance and thermal stability [13]. Therefore, assessing the prophylactic and therapeutic performance of phage X5 against chicken *Salmonella pullorum* infection and exploring viable alternatives to antibiotics under antibiotic-restricted production conditions can offer new clinical insights and generate preliminary pre-clinical evidence. Further dedicated safety assessments and field-scale trials are warranted to rigorously evaluate the efficacy and safety of phage X5 prior to its industrial-scale application in commercial poultry production.

The growth of chickens infected with *S. pullorum* is seriously hindered, the growth performance is reduced, and even a large number of chickens die, causing huge economic losses to the chicken industry. The *Salmonella pullorum* challenge dose applied in this study did not induce mortality in SPF chickens, but significantly retarded their growth and markedly reduced final body weight.

Phage administration alleviated infection-triggered growth impairment and restored average body weight in infected chickens. Compared with group M, the chickens in the phage treatment group T showed better growth performance, indicating that phage X5 could promote the growth of chickens and reduce the adverse effects of *Salmonella pullorum* infection on their growth. Moreover, the pathological changes in the chickens of the phage X5 treatment group T were relatively mild, indicating that phage X5 can effectively alleviate the pathological damage caused by *Salmonella pullorum*. In this experiment, the phage X5 was able to increase the height of the villi in the chickens infected with *Salmonella pullorum* and the ratio of villi to crypts. Moreover, phage X5 preserved ileal villus architecture, which is critical for maintaining intestinal-barrier integrity and supporting anti-infection defenses in young chickens. After *Salmonella* colonized the intestines of chickens, it stimulated the body to produce inflammatory response, increased the expression level of inflammatory cytokines, and destroyed the intestinal morphology [26]. In this study, the expression levels of IL-6 and IL-1β genes in group M were significantly increased, and the obvious changes in intestinal morphology might be related to the colonization of *Salmonella pullorum*.

Oral delivery represents a practical route for treating gastrointestinal bacterial infections. Nevertheless, oral phage therapy is subject to multiple interfering factors, which may lead to unsatisfactory therapeutic outcomes and poor controllability [27]. The intestinal epithelial cell tight junction proteins play a crucial role in the intestinal mucosal barrier. These tight-junction proteins modulate intercellular gaps to maintain appropriate intestinal barrier permeability. Disruption of intestinal epithelial tight-junctions elevates paracellular permeability. This enables pathogenic bacteria to cross the intestinal mucosal barrier, disseminate to internal tissues and organs or enter the bloodstream, and consequently trigger systemic disease [28]. In this study, the phage X5 was able to improve and maintain the morphology and structure of intestinal villi, increase the height of villi in chickens infected with *Salmonella pullorum*, as well as the ratio of villus height to crypt depth, and enhance the expression level of tight junction proteins. It played an important role in maintaining the integrity of the intestinal barrier and in the anti-infection process of chickens.

The detection results of ileum tissue in group M showed a significant upregulation in the mRNA expression levels of IL-1β and IL-6 genes, which may be related to the inflammatory response and intestinal damage caused by *Salmonella pullorum* infection. *Salmonella pullorum* infection in chickens can disrupt the integrity of intestinal morphology, leading to increased expression levels of inflammatory cytokines [26]. Phages possess anti-inflammatory potential [29]; they can not only lyse pathogenic bacteria but also suppress inflammation by modulating innate immune responses including phagocytosis and cytokine production [30]. The levels of *IL-1β*, *IL-6* and *TNF-α* in the serum of chickens in group P and group M significantly decreased, indicating that the phages reduced the inflammatory response. The chickens in the phage X5 treatment group T showed milder clinical symptoms and less severe pathological changes after being infected with *Salmonella pullorum*. This suggests that phage X5 can effectively alleviate the pathological damage caused by *Salmonella pullorum*. In the treatment experiment, amoxicillin, a conventional antimicrobial agent commonly used to control pullorum disease in poultry, was selected as the intervention for positive-control group A. Amoxicillin was given at a constant dose of 0.5 mg per bird instead of a body-weight-scaled mg/kg dose. Fixed-dose dosing mimics real-world commercial poultry production conditions, in which individual chickens are rarely weighed prior to treatment.

In this study, after phage administration, alpha diversity analysis showed that the ACE, Shannon, and Chao indices in group P were significantly higher than those in group M, indicating that group P had a greater total number of cecal microbial species and higher microbial diversity compared to group M. This reflects the changes in cecal microbiota induced by phages. Beta diversity analysis showed that group M was dispersed relative to the C, P, T, and group A, indicating significant differences in cecal microbiota composition between group M and the other groups following phage or antibiotic treatments. This may be related to the lower structural distribution, stability, and maturity of cecal microbiota in young chickens [31]. Videnska classified the evolution of the cecal microbiota of Roman egg chickens into four periods: the first period from hatching to 7 days post-hatching, the second period from the 2nd week to the 4th week, the third period from 2 months of age to 6 months of age, and the fourth period from 7 months of age and above [32]. The main feature of the first period was dominated by bacteria belonging to the *Proteobacteria* class and *Enterobacteriaceae* family; in the second period, *Ruminococcaceae* and *Lachnospiraceae* were the dominant families within the phylum Firmicutes, which is largely consistent with our sequencing results. The first two periods during which chicken cecal microbiota composition was highly variable, and they were also the crucial periods for precisely regulating the distribution and composition of the microbial community [33,34]. In this experiment, both the challenge with the pathogen and the administration of the phage were conducted during the first period. This might be the reason for the significant changes in the microbiota of the cecum. Another study has shown that the diversity and abundance of the intestinal microbiota in chickens after hatching are relatively low [35]. The intestinal microbiota of chickens living in different altitudes and different regions show significant differences, and each has its own unique microbiota [36]. The absence of a phage-only group limits our ability to separate phage-intrinsic effects from infection-rescue effects, and dedicated independent safety evaluation trials are required before real clinical application.

At the family level, analysis of cecal microbiota revealed that the average relative abundances of *Ruminococcaceae* in groups M, P, and T were 18.03%, 20.80%, and 16.71%, respectively, while those of *Lactobacillaceae* were 1.87%, 2.05%, and 1.16%, respectively. Group P exhibited higher relative abundances of both *Ruminococcaceae* and *Lactobacillaceae* compared with group M. These compositional shifts suggest that phage X5 treatment was associated with partial restoration of key commensal bacterial populations in infected chickens. The average relative abundances of the *Lachnospiraceae* family in group M, group P, and group T were 20.11%, 19.19%, and 12.50% respectively. However, it should be noted that 16S rRNA amplicon sequencing only reveals relative compositional changes; the observed abundance alterations do not by themselves constitute functional evidence that these taxa exert beneficial effects, nor do they prove that phage X5 directly improves intestinal health through microbiota remodeling. Functional validation via metagenomics, metabolomics, or culture-based approaches would be required to establish causal biological roles. In the *Lachnospiraceae* family under the Firmicutes phylum, the *Lachnospira* genus has a strong ability to metabolize pectin in fresh fruits or green vegetables and the butyric acid and acetic acid produced by its metabolism serve as important energy sources for the body [37,38].

The relative abundances of *Ruminococcaceae* and *Lactobacillaceae* in group P were higher than those in the untreated infected model group M, indicating a modulatory effect of phage X5 on the intestinal commensal community.

Our results are in line with earlier work reporting that phage CKT1 markedly decreased Salmonella burdens and enhanced the abundances of beneficial Firmicutes taxa such as *Lachnoclostridium*, *Ruminococcus* and *Lactobacillus* [39]. Compared with the previously published CKT1 study, this study set up prevention, treatment and amoxicillin antibiotic positive control groups, and also added intestinal barrier detection. The relative abundance of the Butyricicoccus genus in group P was significantly higher than that in group M. This is a type of bacteria that produces butyric acid, which serves as the main nutrient for intestinal epithelial cells [40,41]. The genus Alistipes is a relatively new bacterial genus that has dual functions of regulating inflammation and influencing metabolism [42]. The relative abundance of the rare Subdoligranulum genus in group P was significantly higher than that in group M. Studies have shown that the Subdoligranulum bacteria maintain intestinal health by producing organic acids and vitamins [43]. The relative abundance of the *Ruminococcus* torques_group genus in group P showed an upward trend compared to group M, but there was no significant difference. The rumen bacteria genus can ferment and break down cellulose, capable of degrading cellulose into glucose and other sugars, and through metabolism produce short-chain fatty acids such as acetic acid and propionic acid, providing nutrients for intestinal epithelial cells [44]. The changes observed in the composition of the microbial community in this study are relevant findings, but their functional significance needs to be determined in future research. This study collected samples only at the terminal time point. Dynamic sampling across multiple time points and further targeted validation experiments are needed to confirm causality.

To overcome the limitation of narrow phage–host range, researchers have proposed developing bacteriophage cocktail preparations. This strategy combines multiple individual phage preparations with distinct host specificities [45]. Each type of single-bacterium formulation plays a specific role in the “cocktail” for targeting a particular *Salmonella* serotype or strain, thereby collectively expanding the host range of the entire formulation [46]. The broad lysis spectrum phage X5 used in this study has shown excellent efficacy in preventing and treating chicken white diarrhea caused by *Salmonella pullorum*. It has great potential and value for developing *Salmonella pullorum* phage “cocktail” therapy and clinical applications. One limitation of the present study is that serial sampling across multiple time points post-challenge was not performed. In subsequent experiments, more detailed sampling time points will be adopted. All the experiments were conducted in strictly controlled SPF isolation chambers, which lacked the complex microbial background, environmental stress and mixed infection pressure present in commercial poultry farms. Additionally, there was no monitoring of the emergence of phage-resistant *Salmonella pullorum* variants during the intervention period. Residual active phages in the tissue homogenate may cause in vitro lysis of *S. pullorum* during sample processing and plating, thereby artificially reducing the recovered CFU count. Although serial dilution has been used to reduce phage concentration, this step cannot completely eliminate this potential bias. Future studies should further investigate the application effect of phage X5 under commercial chicken conditions, systematically evaluate its in vivo survival and proliferation kinetics, and long-term track the emergence of resistant strains and the co-evolution of phage–host, in order to comprehensively assess the application potential and safety of phage therapy.

This study describes the potential related pathways by which phage X5 alleviates growth impairment in chickens, which involves inhibiting pathogenic bacteria, improving the intestinal microecology, and suppressing inflammatory responses. This study evaluated the preventive effect of phage X5 on the infection of *Salmonella pullorum* in chickens, providing initial clues to understanding the protective effects of phage X5. The guidance suggestion given is that prophylactic administration of phages can alleviate or reduce the damage caused by the infection of *Salmonella pullorum*. Nevertheless, given that sampling in the present study was restricted to a single time point, definitive causal relationships between these interconnected biological events cannot be rigorously established. Further studies incorporating multi-time-point sampling and targeted functional validation are therefore warranted to unravel the precise in vivo mechanisms behind phage X5-mediated host protection.

## 5. Conclusions

This study confirmed that phage X5 could reduce the *Salmonella pullorum* load in chickens, alleviate the growth inhibition and intestinal inflammation caused by infection, and partially restore the cecal microbiota, which provided a preliminary in vivo basis for the phage biological control of *Salmonella pullorum*.

## Figures and Tables

**Table 1:** Gene primer sequence.

Gene	Primer Sequence
*IL-1β*	GTGAGGCTCAACATTGCGCTGTA
	TGTCCAGGCGGTAGAAGATGAAG
*IL-6*	AATCCCTCCTCGCCAATCT
	TCACGGTCTTCTCCATAAACG
*IFN-γ*	ACACTGACAAGTCAAAGCCGC
	AGTCGTTCATCGGGAGCTTG
*Claudin-3*	AGCCCTCCATCTCAGCAG
	TTCTCCGCCAGACTCTCC
*Occludin*	TGTGTAAGGCCCACACCTCT
	TGCTCAGGGTACCATTCTGG
*ZO-1*	CTTCAGGTGTTTCTCTTCCTCCTC
	CTGTGGTTTCATGGCTGGATC
*β-actin*	TTGTATCTTCCGCCTTAA
	CCCACATACTGGCACTT

## Data Availability

The datasets presented in this study can be found in online repositories. The names of the repository/repositories and accession number(s) can be found below: NCBI: PRJNA1524118.
